# Nerol as an anti-quorum sensing and therapeutic agent against *Acinetobacter baumannii* pneumonia

**DOI:** 10.1016/j.isci.2026.115696

**Published:** 2026-04-13

**Authors:** Qing Lu, Shuyun Wei, Yu Sun, Zhen Liang, Yulong Li, Hong Zeng

**Affiliations:** 1 Guangxi Technology Innovation Cooperation Base of Prevention and Control Pathogenic Microbes With Drug Resistance, Youjiang Medical University for Nationalities, Baise, Guangxi 533000, P.R. China; 2 Guangxi Zhuang Autonomous Region Engineering Research Center of Clinical Prevention and Control Technology and Leading Drug for Microorganisms with Drug Resistance in Border Ethnic Areas, Baise, Guangxi 533000, P.R. China

**Keywords:** biological sciences, microbiology

## Abstract

*Acinetobacter baumannii* is a major nosocomial pathogen causing pneumonia; its virulence, biofilm formation, and antibiotic resistance are all regulated by quorum sensing (QS). Nerol, a monoterpene derived from orange peel, exhibits antibacterial activity. This study demonstrates that Nerol exhibits a minimum inhibitory concentration (MIC_90_) of 0.5 mg/mL against *A. baumannii*. At subinhibitory concentrations, it inhibits N-acyl-homoserine lactones, biofilm formation, motility, and extracellular polymeric substance (EPS) production. Proteomics revealed synchronous downregulation of virulence proteins, including BfmS, YiaD_1, MacB, MurF, and MtgA. ITC confirmed its 2:1 stoichiometric, exothermic binding to the BfmS sensor domain (KD 1.3 × 10^−4^ M), disrupting the BfmRS two-component system and blocking downstream QS pathways. Experiments demonstrated that Nerol significantly reduced the gene expression and protein secretion levels of proinflammatory cytokines (TNF-α, IL-6, and IL-1β) by inhibiting the activation of the NF-κB/MAPK signaling cascade. Nerol’s ability to counteract QS and alleviate inflammatory responses highlights its potential as a therapeutic agent for treating *A. baumannii* infections.

## Highlights

• Nerol inhibits quorum sensing and biofilm formation in *A. baumannii*
• Nerol disrupts the quorum-sensing pathway through the BfmRS system
• Nerol downregulates the expression of pathogenicity factors in *A. baumannii*
• Nerol demonstrates therapeutic efficacy against *A. baumannii* pneumonia *in vivo*

## Graphical abstract

**Figure:**
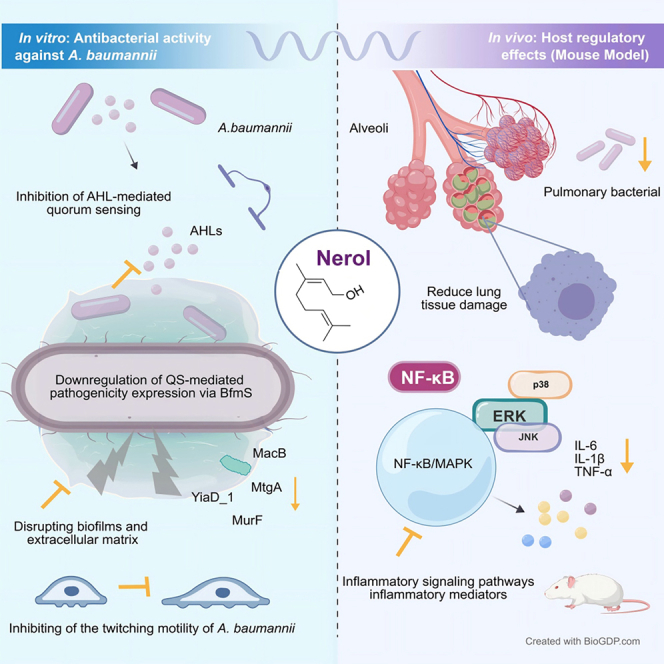

## Introduction

*Acinetobacter baumannii* is a highly virulent opportunistic pathogen, commonly causing bloodstream infections, pneumonia^1^ and meningitis.^2^ Recognized as a leading ESKAPE pathogen in healthcare settings, it is classified by the World Health Organization (WHO) as a critical priority pathogen.^3^ Antibiotic resistance and adaptation to desiccation in *A. baumannii* are significant factors in the development of nosocomial infections.^4^^,^^5^ Current data indicate an overall mortality rate of 43% in ICU patients with *A. baumannii* infection, posing substantial therapeutic challenges.^6^ According to the China Antimicrobial Surveillance Network (CHINET), carbapenem resistance (imipenem/meropenem (MEM)) in *A. baumannii* isolates remained stable (>75%) from 2018 to 2022.^7^ In addition, Wang et al.^8^ analyzed 255 clinical samples collected from Youjiang Medical University for Nationalities, finding that 71.2% were resistant to MEM and 68.6% were resistant to imipenem (IPM). Resistance mechanisms include drug-inactivating enzymes, altered drug targets, enhanced outer membrane permeability, biofilm formation, and efflux pump overexpression. Notably, virulence factors such as efflux pumps, lipopolysaccharides, and outer membrane protein A (OmpA) facilitate bacterial adhesion to epithelial cells and provoke host immune responses.^9^ Therefore, the search for antimicrobial agents capable of inhibiting efflux pumps, biofilm formation, and virulence factors is crucial. The BfmRS two-component system (TCS) regulates virulence factor expression, antimicrobial resistance, and environmental adaptation, with BfmR acting as a response regulator that pairs with BfmS to control virulence genes.^10^ Studies have shown that BfmS-deficient mutants exhibit reduced biofilm formation and twitching motility, while BfmR enhances resistance to certain antimicrobial agents and has been identified as a critical therapeutic target *in vivo.*^10^^,^^11^^,^^12^ Furthermore, OmpA, an abundant surface protein, plays a critical role in pathogenesis. Its C-terminal domain anchors to the cell wall via non-covalent peptidoglycan binding at conserved amino acid sites.^13^ Additionally, *A. baumannii* possesses a unique quorum sensing (QS) system comprising the autoinducer synthase (AbaI) and the autoinducer receptor (AbaR). This system regulates biofilm formation and virulence through N-acyl-homoserine lactone (AHL) signaling molecules. Upon reaching threshold concentrations, these molecules activate pathogenic functions.^14^ To date, a spectrum of QS signals—AHLs, the diffusion signal factor (DSF) family, and autoinducer-2 (AI-2)—has been characterized. These systems orchestrate coordinated bacterial adaptation to environmental shifts, often culminating in enhanced drug resistance and virulence.^15^ In *A. baumannii*, AbaI predominantly synthesizes 3-OH-C12-HSL, yet culture supernatants also contain unsubstituted C10-HSL, C12-HSL, and C14-HSL, revealing broad AbaI substrate specificity.^16^ The AbaI/AbaR pair governs biofilm architecture and twitching motility, while AHLs signaling additionally modulates outer-membrane protein A, efflux pumps, and Type I pili, underscoring its central role in *A. baumannii* pathogenicity.^17^

Nerol, a monoterpene alcohol originating from orange peel (*Citrus reticulata*), was documented as “Chen Pi” in the Compendium of Materia Medica for relieving “chest obstruction and cough.”^18^ Traditionally combined with Pinellia ternata (Ban Xia) and Poria cocos (Fu Ling) to dispel wind-cold and resolve phlegm, its volatile oils show potential against respiratory infections. Phytochemical studies identify key components, including limonene, α-Pinene, and Nerol. Nerol exhibits diverse pharmacological activities: antimicrobial effects against Gram-positive/negative bacteria,^19^ antioxidant capacity,^20^ and potential antitumor efficacy via apoptosis induction.^21^ However, systematic studies on Nerol’s activity against *A. baumannii* remain lacking.

This study used microdilution assays, AHLs reporter strain colorimetric detection, and quantitative reverse transcription polymerase chain reaction (RT-qPCR) techniques to investigate the inhibitory activity of Nerol against the QS system of *A. baumannii*. The mechanism of action was then elucidated through biofilm disruption, motility assays, quantification of the extracellular polymeric substance (EPS), and proteomics analysis. Ultimately, in a mouse pneumonia model, Nerol demonstrated *in vivo* efficacy by reducing the bacterial load in the lungs, suppressing inflammatory mediators, and repairing tissue damage.

## Results

### Nerol exerts inhibitory activity against *A. baumannii*

**Figure 1:**
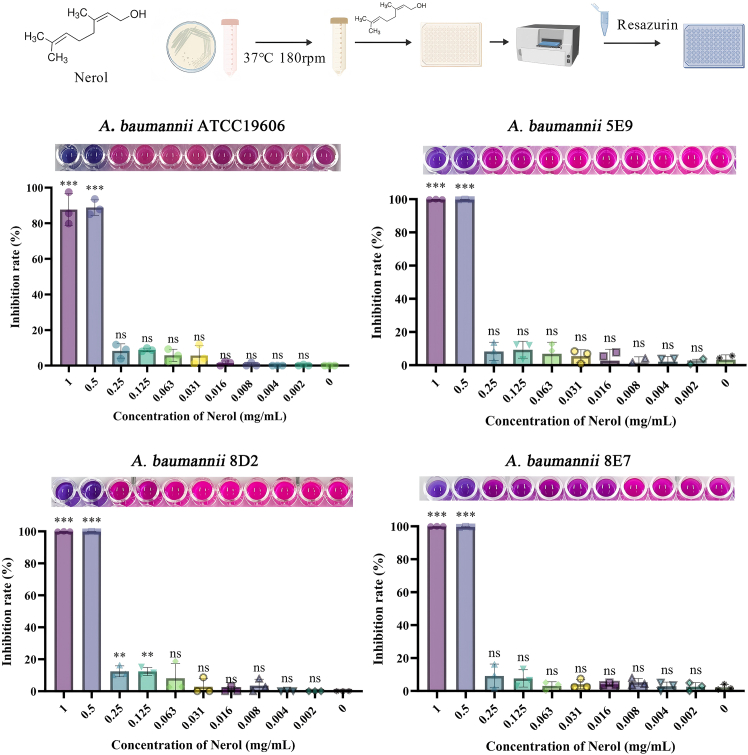
MIC of Nerol against *A. baumannii* The flowchart was created by https://biogdp.com/. Data are displayed as the means ± SD from three independent experiments, and significance was determined by one-way ANOVA with Dunnett’s post-test vs. Control: ∗*p* < 0.05, ∗∗*p* < 0.01, and ∗∗∗*p* < 0.001; ns, *p* ≥ 0.05. The remaining unshown strains are listed in Figure S1.

Nerol exhibits consistent similar activity inhibition against both one standard strain *A. baumannii* ATCC 19606 and 11 clinical isolates strain with a minimum inhibitory concentration (MIC) at 0.5 mg/mL (Figures 1 and S1). As shown in Figure S2, at MIC concentrations, Nerol significantly inhibited the growth of *A. baumannii*. In the 1/2MIC, 1/4MIC, and control groups, *A. baumannii* exhibited similar logarithmic growth trends within 24 h, indicating that Nerol had no inhibitory effect on the growth of the strains. At a Nerol concentration of 1/2MIC, floating colony-forming units (CFUs) remained ≥90% of untreated controls. Notably, 0.1% (v/v) Tween-80 (vehicle) did not affect growth or virulence: CFU recovery was ≥97% for all strains (*p* > 0.05, Table S1). Among the clinical isolates tested, strains *A. baumannii* 5E9 and *A. baumannii* 8D2 were identified as extensively drug-resistant (XDR) strains, which exhibited resistance to cephalosporins, carbapenems, quinolones, and β-lactam/β-lactamase inhibitor combinations. And the strain *A. baumannii* 8E7 was a multidrug-resistant (MDR) strain: it was resistant to carbapenems, cephalosporins, β-lactam/inhibitor combinations, and sulfonamides, while remaining susceptible to quinolones (Table S2). Additionally, strains *A. baumannii* 5E9, *A. baumannii* 8D2, and *A. baumannii* 8E7 possess the property of forming strongly positive biofilms.^8^ These strains encompass two typical drug-resistant phenotypes (XDR and MDR) and exhibit distinct antimicrobial resistance profiles. Therefore, strains *A. baumannii* 5E9, *A. baumannii* 8D2, and *A. baumannii* 8E7 were selected for subsequent studies on antimicrobial activity verification and drug resistance mechanisms.

### Nerol inhibits quorum-sensing-regulated phenotypes in *Chromobacterium violaceum*

The biosensor strain *C. violaceum* ATCC 12472 and CV026, which has been widely used to investigate anti-QS activity of the most active constituents.^14^^,^^22^ To evaluate the anti-quorum-sensing potential of Nerol, we first determined its MIC against *C. violaceum* ATCC 12472 and CV026, which was 0.125 mg/mL (Figure S3A). At this MIC, Nerol inhibited bacterial growth, while 1/2 and 1/4MIC and the control group exhibited typical logarithmic growth over 24 h (Figure S3B).

At 1/2MIC (0.0625 mg/mL), purple pigment production decreased by 80% (80.19 ± 0.37, *p* < 0.05, Figure S3C), while planktonic growth remained ≥90% of the untreated control, indicating pigment loss was not caused by cell death. At the same 1/2MIC concentration, twitching motility decreased by 44.95% ± 2.56% (Figure S3D). Biofilm crystal violet quantification reduced by 89.29% ± 2.8% (Figure S3E), accompanied by loss of dense structural features. Consistent with the quantitative data, microscopic observation confirmed that Nerol treatment disrupted the dense, structured architecture of the biofilms compared to the control group.

Collectively, these results demonstrate that Nerol, at sub-inhibitory concentrations, effectively disrupts the QS system of *C. violaceum*, attenuating the production of violacein, twitching motility, and biofilm formation—key virulence traits controlled by QS.

### Inhibitory effect of Nerol on the QS system of *A. baumannii*

#### Inhibition of AHLs synthesis by Nerol in *A. baumannii*

**Figure 2:**
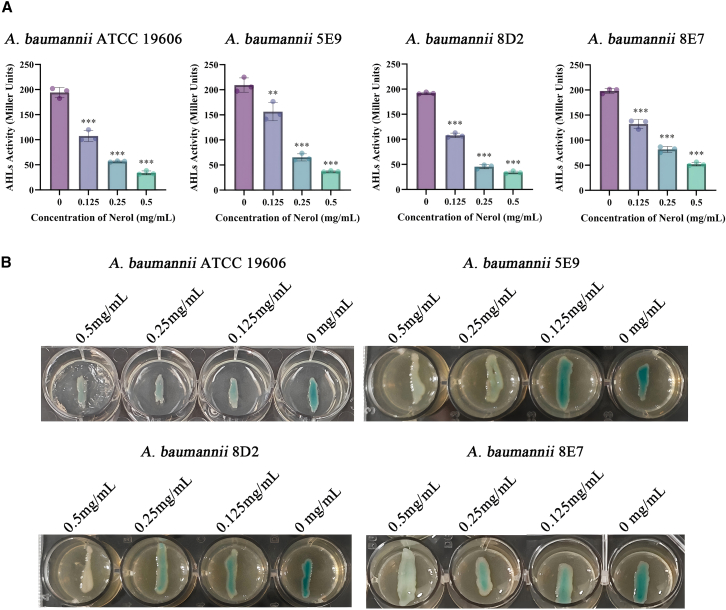
Inhibition of AHLs signaling molecules in *A. baumannii* by Nerol (A) Quantitative Approach: Using *A. tumefaciens* KYC55 as an indicator strain, AHLs-induced β-galactosidase hydrolyses ONPG to produce a yellow product. After treating *A. baumannii* with 0, 0.125, 0.25, or 0.5 mg/mL Nerol, the supernatant was incubated with KYC55, and AHLs activity was assessed by measuring OD_420_. (B) Plate assay: Following the same principle, X-gal served as the substrate. KYC55 colonies turned blue in the presence of AHLs; color intensity indicated relative AHLs content. Data are displayed as the means ± SD from three independent experiments, and significance was determined by one-way ANOVA with Dunnett’s post-test vs. Control: ∗*p* < 0.05, ∗∗*p* < 0.01, and ∗∗∗*p* < 0.001; ns, *p* ≥ 0.05.

The biofilm formation of *A. baumannii* is regulated by its QS pathway, in which AHLs function as the primary signaling molecules.^23^ We utilized the *Agrobacterium tumefaciens* KYC55 reporter strain, which produces a colorimetric response, to quantify AHLs production (Figure 2A).

The results indicated that Nerol inhibited AHLs synthesis in a concentration-dependent manner. In strain ATCC 19606, Nerol reduced AHLs levels by 44.6%, 70.8%, and 82.5% at concentrations of 0.143, 0.285, and 0.5 mg/mL, respectively. A comparable inhibitory effect on AHLs production was observed in the clinical isolates (5E9, 8D2, and 8E7) across the same concentration gradients.

The most pronounced inhibition was observed in strains 5E9 and 8D2, which showed a reduction in AHL activity of up to 82.3% at 0.5 mg/mL. This concentration-dependent inhibition was further corroborated by agar plate diffusion assays, where the blue color intensity of the *A. tumefaciens* KYC55 reporter gradually diminished with increasing Nerol concentrations, indicating suppressed AHLs production (Figure 2B). Further quantitative results from high-performance liquid chromatography (HPLC) analysis revealed that at 1/2 MIC concentration, HPLC showed a 78.9% reduction in AHLs (Table S3), consistent with the 82.3% decrease observed in the KYC55 reference strain.

Collectively, these findings demonstrate that Nerol dose-dependently suppresses AHLs synthesis in *A. baumannii*, thereby interfering with QS-mediated signaling.

### Prediction of Nerol binding affinity to AbaR, AbaI, BfmR, and BfmS via molecular docking

**Figure 3:**
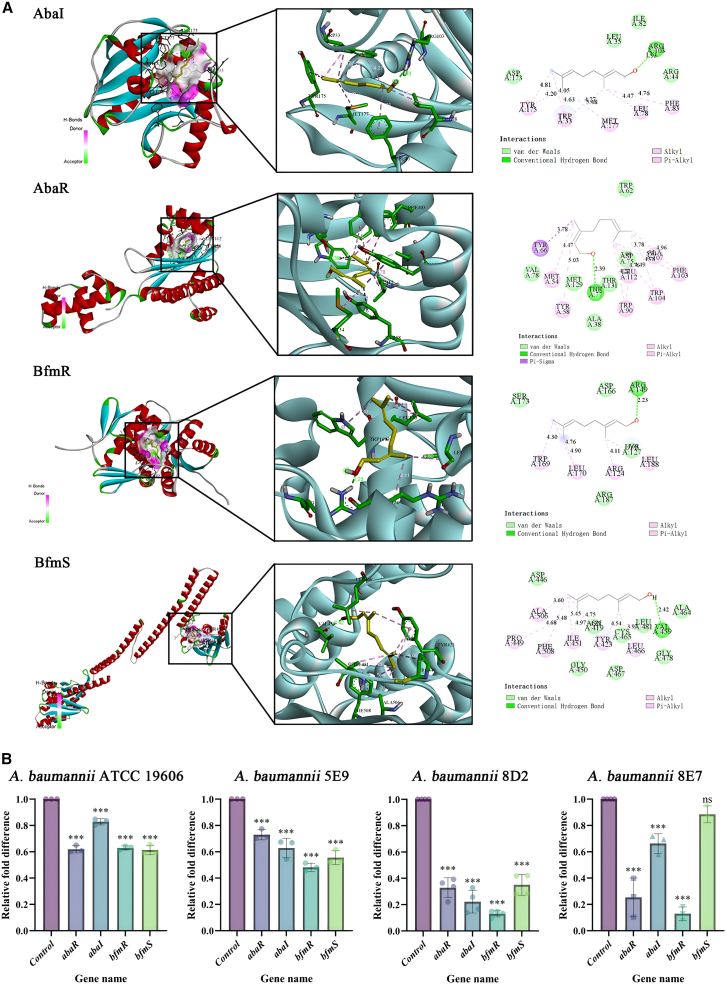
Nerol downregulates key genes in the two-component QS system of *A. baumannii* (A) Molecular docking: displays two-dimensional interaction maps and three-dimensional binding conformations of Nerol with AbaI, AbaR, BfmS, and BfmR. (B) RT-qPCR: Relative expression levels of *abaI*, *abaR*, *bfmS*, and *bfmR* mRNA following Nerol treatment (0.25 mg/mL), measured using the 2^−ΔΔCt^ method with 16S rRNA as internal control. Data are displayed as the means ± SD from three independent experiments, and significance was determined by one-way ANOVA with Dunnett’s post-test vs. Control: ∗*p* < 0.05, ∗∗*p* < 0.01, and ∗∗∗*p* < 0.001; ns, *p* ≥ 0.05.

First, molecular docking simulations predicted favorable binding energies for Nerol with the target QS-related proteins, including AbaI, AbaR, BfmR, and BfmS, and the binding energies were −5.4, −6.0, −5.1, and −5.7 kcal/mol. The analysis of the specific binding modes revealed that Nerol engages with key residues of each protein: For AbaR, Nerol formed a hydrogen bond with Thr77, hydrophobic interactions with multiple residues (e.g., Met54 and Tyr58), and van der Waals forces with others (e.g., Val78 and Asp75). Similarly, Nerol binding to AbaI involved a hydrogen bond with Arg44, alongside hydrophobic interactions with residues such as Tyr175 and Trp33. For BfmR, a hydrogen bond was predicted with Arg149, complemented by hydrophobic interactions with Trp169 and Leu170. Finally, for BfmS, Nerol was predicted to form a hydrogen bond with Val459 and hydrophobic interactions with Pro449 and Phe508, among others (Figure 3A).

### Nerol reduces AHLs synthesis in *A. baumannii* by inhibiting *abaR*, *abaI*, *bfmR,* and *bfmS* expression

Further, RT-qPCR analysis revealed that Nerol treatment significantly suppressed both the AbaI/AbaR and BfmRS dual-component systems regulating AHLs synthesis in *A. baumannii*. Transcriptional levels of the target genes *abaI*, *abaR*, *bfmR*, and *bfmS* were downregulated, with reductions ranging from 11.57% to 87.03% (Figure 3B). In strain *A. baumannii* 8D2, transcription levels of all four genes decreased by over 60% (*p* < 0.05), with *bfmR* downregulated by 86.93%; strain *A. baumannii* 8E7 also exhibited significant suppression of *bfmR* expression (87.03%, *p* < 0.05). In addition, strains *A. baumannii* 5E9 and the standard strain ATCC 19606 showed varying degrees of transcriptional suppression, consistent with the decreasing AHL concentrations in the *A. baumannii* culture supernatant.

### Interference of Nerol with biofilm formation in *A. baumannii*

**Figure 4:**
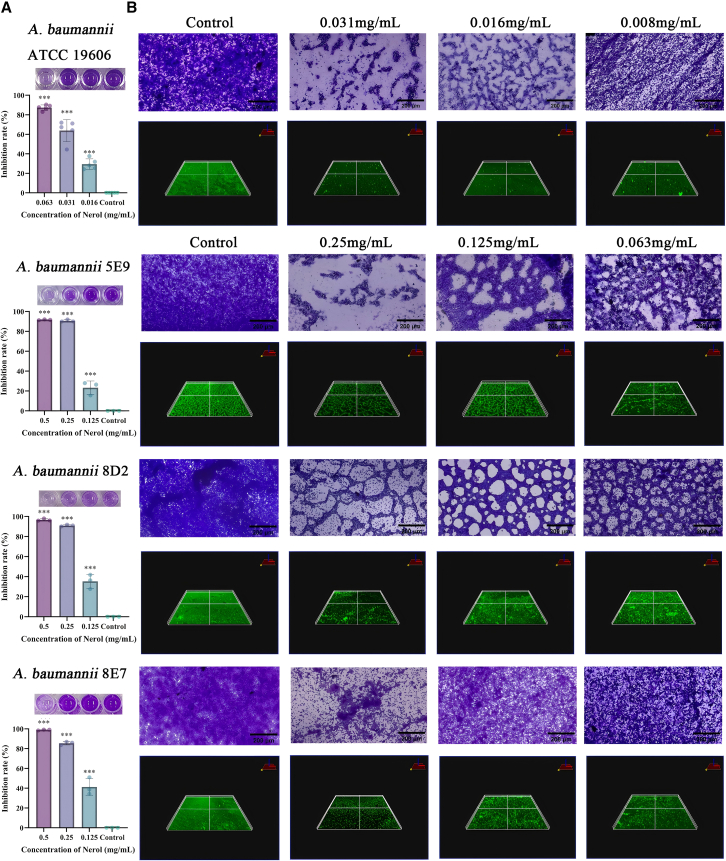
Nerol inhibits biofilm formation in *A. baumannii* (A) Quantitative results of Nerol on biofilm formation of *A. baumannii*. (B) Morphological observation by crystal violet staining and biofilm thickness by confocal microscopy. Scale bars, 200 μm. Data are displayed as the means ± SD from three independent experiments, and significance was determined by one-way ANOVA with Dunnett’s post-test vs. Control: ∗*p* < 0.05, ∗∗*p* < 0.01, and ∗∗∗*p* < 0.001; ns, *p* ≥ 0.05.

The minimum biofilm inhibitory concentration (MBIC) of Nerol against *A. baumannii* was first determined. Against the standard strain ATCC 19606, Nerol exhibited an MBIC of 0.031 mg/mL, resulting in 63.7% inhibition of biofilm formation (information on biofilm formation capacity is shown in Table S4). In contrast, the clinical isolates (5E9, 8D2, and 8E7) shared a higher common MBIC of 0.25 mg/mL. At this concentration, Nerol strongly inhibited biofilm formation by 90.6%, 90.9%, and 85.5% in strains 5E9, 8D2, and 8E7, respectively (Figure 4A).

To further characterize Nerol-induced changes in biofilm structure, light microscopy and confocal laser scanning microscopy (CLSM) were used to visualize mature biofilms. Untreated control biofilms displayed a robust, three-dimensional architecture with high density, multiple layers, and extensive bacterial aggregation. In contrast, treatment with Nerol at the MBIC markedly disrupted biofilm morphology across all strains, leading to cell dispersal and a noticeable thinning of the extracellular matrix. The analysis of CLSM 3D reconstructions confirmed significant reductions in both biofilm biomass and average thickness compared to the untreated control (Figure 4B). These findings demonstrate that Nerol, at the MBIC, effectively disrupts *A. baumannii* biofilms by impairing their structural integrity and reducing matrix thickness.

### Nerol reduces the extracellular polymer content of *A. baumannii*

**Figure 5:**
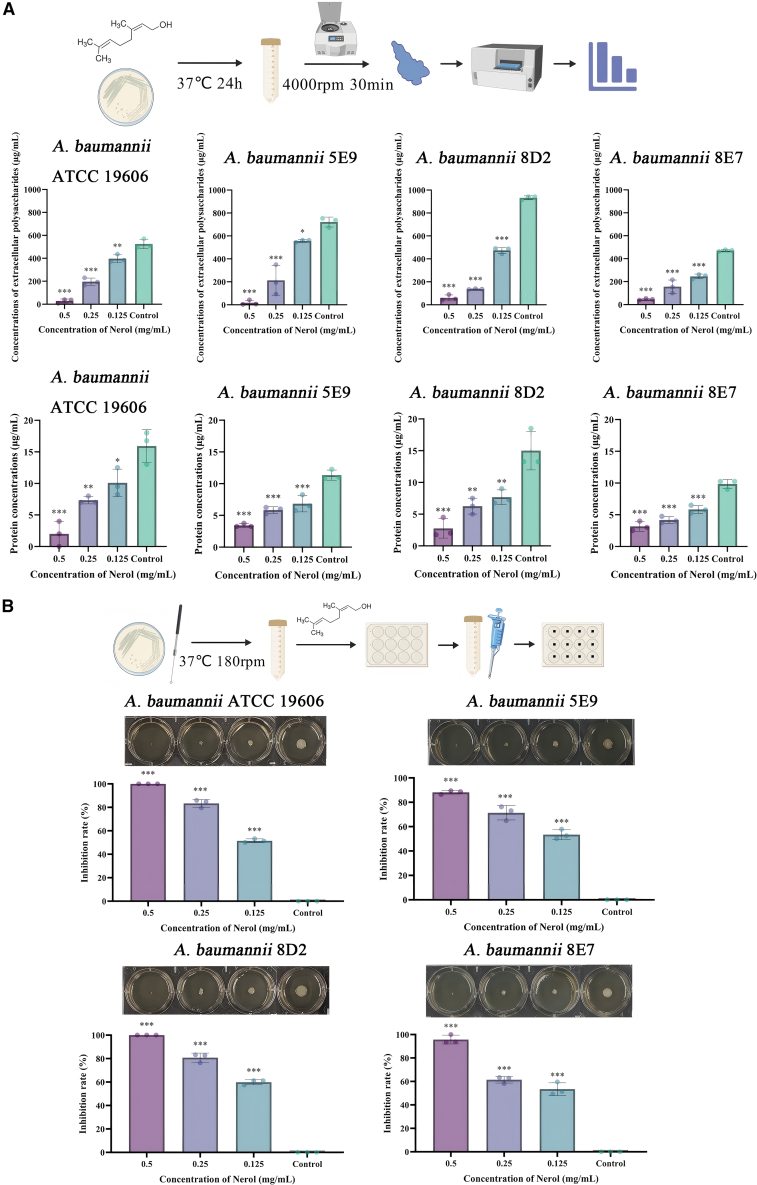
Nerol reduces EPS content and inhibits twitching motility in *A. baumannii* (A) EPS quantification: Following 24 h treatment with Nerol (0, 0.125, 0.25, 0.5 mg/mL), extracellular proteins were measured using the Bradford method and extracellular polysaccharides via the phenol-sulphate assay; the flowchart was created by https://biogdp.com/. (B) Twitching motility: 5 μL of bacterial suspension (1.0 × 10^8^ CFU/mL) was spotted centrally on LB agar plates and incubated statically. Migration diameter (mm) was measured perpendicularly at the cross, and the inhibition rate was calculated. Data are displayed as the means ± SD from three independent experiments, and significance was determined by one-way ANOVA with Dunnett’s post-test vs. Control: ∗*p* < 0.05, ∗∗*p* < 0.01, and ∗∗∗*p* < 0.001; ns, *p* ≥ 0.05.

The biofilm matrix, primarily composed of EPS and proteins, forms a protective barrier that enhances bacterial tolerance to antimicrobial agents.^24^ Quantitative analysis revealed that Nerol treatment significantly and concentration-dependently reduced the extracellular polysaccharide and protein content of the biofilms compared to the untreated control. At a concentration of 0.25 mg/mL, Nerol reduced protein levels by 1.94- to 2.4-fold and polysaccharide levels by 2.68- to 6.82-fold across all tested strains (ATCC 19606, 5E9, 8D2, 8E7) compared to the control (*p* < 0.05, Figure 5A).

These results indicate that Nerol effectively inhibits the production of EPS in *A. baumannii* biofilms, as demonstrated by the concomitant reduction in both polysaccharide and protein matrix components.

### Nerol reduces twitching motility in *A. baumannii*

Twitching motility in *A. baumannii*, a behavior mediated by type IV pili (TFP), was significantly inhibited by Nerol. At a concentration of 0.25 mg/mL, Nerol reduced twitching motility by 61.4%–83.4% across all tested strains (83.4% in ATCC 19606, 71.4% in 5E9, 80.8% in 8D2, and 61.4% in 8E7) compared to the control (Figure 5B).

This impairment of the TFP-dependent twitching motility is consistent with Nerol’s previously demonstrated disruption of QS pathways. By reducing AHLs synthesis and interrupting QS signaling, Nerol consequently suppresses multiple QS-regulated virulence traits, such as twitching motility, biofilm formation, and EPS secretion.

### Treatment with Nerol significantly reduces the expression of proteins related to virulence factors in *A. baumannii*

**Figure 6:**
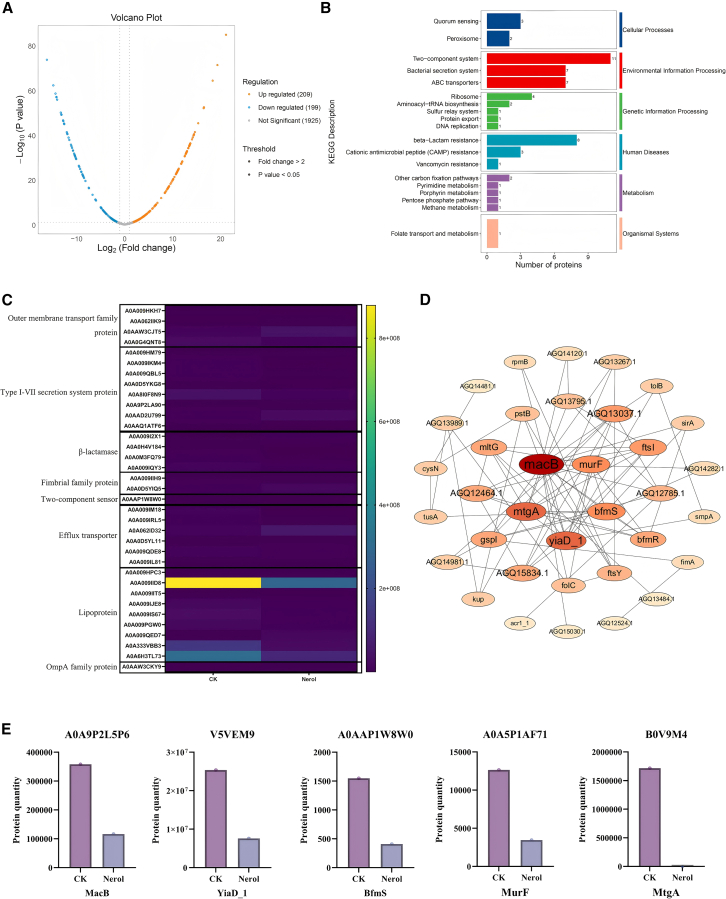
Nerol down-regulates the expression of virulence-related proteins in *A. baumannii* (A) Differential protein volcano map. (B) Differential protein KEGG annotation categorization bar chart. (C) Heatmap of differential protein clustering. (D) Protein interaction network diagram. (E) Difference in expression statistics of key proteins.

To elucidate the molecular mechanism of Nerol’s antibacterial action against *A. baumannii*, we performed a quantitative proteomic analysis using the Astral DIA platform. A volcano plot was generated to visualize differentially expressed proteins between the treatment and control groups. This analysis identified 209 upregulated and 199 downregulated proteins (Figure 6A). KEGG pathway enrichment analysis categorized the differentially expressed proteins, with significant enrichment observed in pathways related to cellular processes, environmental information processing, and human diseases (Figure 6B). These proteins are categorized according to their functions, involvement in secretion, efflux, and membrane-associated activities (Figure 6C). Notably, key virulence-related proteins were substantially downregulated. These included outer membrane proteins (e.g., OmpA, −4.51-fold; YiaD_1, −1.74-fold), efflux pump components of the RND superfamily (−14.20-fold), bacterial secretion system proteins (e.g., J512_2382, −13.28-fold), and the TCS regulator BfmS (−1.92-fold) (Table 1).

**Table 1:** Downregulated proteins of *A. baumannii* exposed to Nerol

Protein	Genes	Accession Number	Functions	Log2(Fold change)
Efflux transporter, RND family	J512_1967	A0A009IM18	Xenobiotic transport out of the cell	−14.20
Type II secretion system protein K	J512_2382	A0A009IKM4	Moving substrates across the cell membrane	−13.28
β-lactamase hydrolase-like protein	Blh_2	A0A0M3FQ79	Inactivating hydrolases of the antibiotic β-lactam ring	−12.56
Biosynthetic peptidoglycan transglycosylase	MtgA	A3M3C7	cell wall organization and peptidoglycan biosynthetic process	−12.04
Outer membrane beta-barrel domain protein	J512_2959	A0A009HKH7	Maintaining membrane permeability, participating in substance transport	−11.91
Trimeric autotransporter adhesin Ata	ATCC19606_28130	A0A6F8THN4	Mediating adhesion between bacteria	−11.67
Type IV secretion-associated protein, family	ABR2091_1301	A0A0D5YKG8	Transporter proteins and ribonucleoprotein complexes	−10.11
OmpA family protein	HMPref. 0010_01571	D0C913	Adhesion, toxicity, invasiveness, and biofilm formation	−4.51
Large ribosomal subunit protein bL28	RpmB	A3M1V9	Catalyze protein synthesis	−2.15
Type IV pilus biogenesis family protein	J506_1850	A0A009QBL5	Involved in biofilm formation, gliding, or twitching movements	−2.00
Two-Component system	BfmS	A0AAP1W8W0	Signaling systems within bacteria that allow organisms to sense and respond to environmental changes	−1.92
UDP-N-acetylmuramoyl-tripeptide--D-alanyl-D-alanine ligase	MurF	A0A5P1AF71	cell wall organization and peptidoglycan biosynthetic process	−1.87
OmpA family protein	YiaD_1	V5VEM9	Adhesion, toxicity, invasiveness, and biofilm formation	−1.74
ABC transporter family protein	MacB	A0A9P2L5P6	transmembrane transporter activity	−1.62

Protein-protein interaction network analysis using STRING v12 revealed significant interconnectivity among the differentially expressed proteins, further underscoring their collective role in signal transduction and transport mechanisms (Figure 6D). The analysis of the interaction network highlighted five key proteins (MacB, YiaD_1, BfmS, MtgA, and MurF) as central nodes involved in regulating virulence through distinct pathways. The changes in the levels of the above proteins before and after Nerol treatment are shown in Figure 6E. Specifically, BfmS (−1.92-fold) is a key regulator of biofilm formation and twitching motility. MacB (−1.62-fold) (Table 1) and the outer membrane pore protein YiaD_1 (−1.74-fold) function as an efflux pump complex that exports antimicrobials and virulence factors. Additionally, MurF (−1.87-fold) and MtgA (−12.04-fold) are essential enzymes for peptidoglycan biosynthesis in the cell wall.

### The sensor domains of Nerol and BfmS have high-affinity multi-site binding characteristics

To corroborate the QS-inhibitory phenotype conferred by Nerol, we next quantified its direct interaction with the BfmS—the only core target common to both our RT-qPCR (transcriptional down-regulation) and proteomic datasets. Isothermal titration calorimetry (ITC) revealed that Nerol binds directly to the sensor domain of BfmS with a 2:1 stoichiometry (*N* = 2.13 ± 0.24) and a sub-millimolar affinity (KD = 130 ± 120 μM). The interaction is strongly exothermic (ΔH = −335 ± 227 kJ/mol) and enthalpy-driven, with a favorable Gibbs free energy (ΔG = −22.2 kJ/mol) and a compensatory entropy contribution (TΔS = 313 kJ/mol) (Figure S4). These thermodynamic signatures indicate that Nerol occupies multiple sites on BfmS via a combination of enthalpic contacts and entropy-driven hydrophobic effects, providing a biophysical basis for the downstream disruption of the BfmRS quorum-sensing cascade.

### Nerol exhibits a favorable safety profile

The biocompatibility and *in vivo* safety of Nerol were assessed using macrophage cytotoxicity and murine oral toxicity models. *In vitro*, CCK-8 assays on RAW264.7 macrophages showed no significant cytotoxicity at Nerol concentrations up to 4 mg/mL (*p* > 0.05; Figure S5). *In vivo* safety was further assessed via oral gavage in mice administered Nerol at doses of 30, 100, and 300 mg/kg. Histopathological examination of liver and spleen tissues harvested on day 7 revealed no structural abnormalities or lesions, even at the highest dose (300 mg/kg), compared with the control group (Figure S5).

Together, these findings indicate that Nerol exhibits minimal cytotoxicity at effective concentrations and shows no evidence of organ toxicity at the administered doses, supporting its potential therapeutic safety.

### Nerol alleviates *A. baumannii* pneumonia in mice by modulating host inflammation

**Figure 7:**
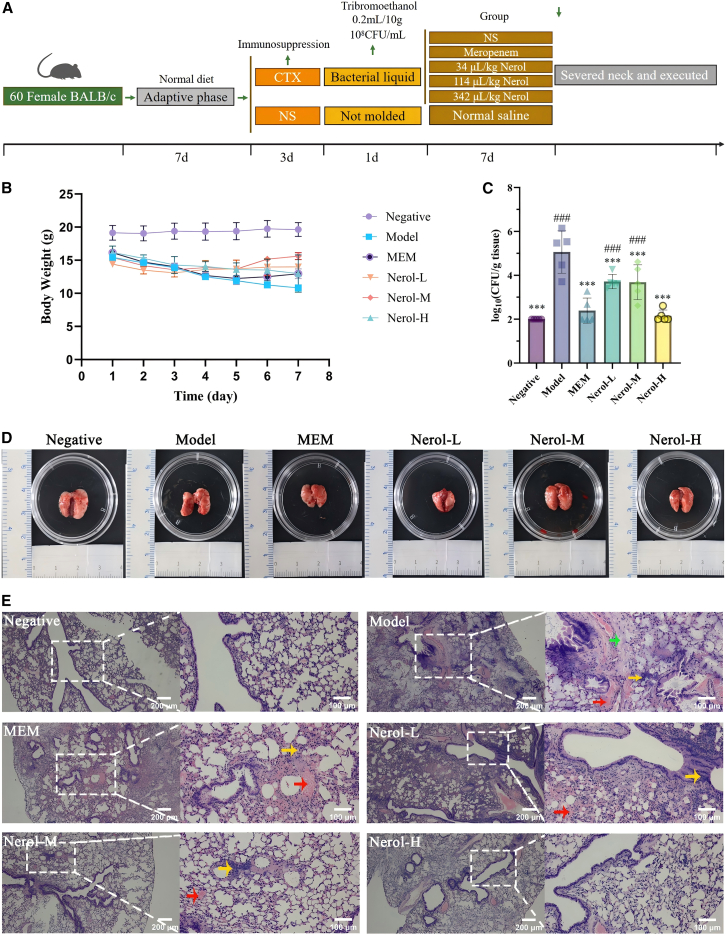
Evaluation of *in vivo* activity of Nerol against pneumonia caused by *A. baumannii* (A) Flowchart of the modeling process of experimental pneumonia in BALB/c mice *in vivo* and the therapeutic use of Nerol. (B) Changes of body weight of mice under different treatment conditions. (C) Changes of bacterial load in the lungs of mice under different treatment conditions; values below the LOD were set to LOD/2 for log-transformation and statistical analysis. (D) Morphology of whole lungs of mice. (E) HE staining of lung tissues of mice (40×: scale bars, 200 μm, 100×: scale bars, 100 μm). Negative: negative group, model: model group, MEM: meropenem group, Nerol-L: Nerol low dose group (30 mg/kg), Nerol-M: Nerol medium dose group (100 mg/kg), Nerol-H: Nerol high dose group (300 mg/kg). Data are displayed as the means ± SD from three independent experiments. Bacterial load was compared with the model group using one-way ANOVA of variance combined with Dunnett’s test: ∗*p* < 0.05, ∗∗*p* < 0.01, and ∗∗∗*p* < 0.001; ns, *p* ≥ 0.05. Compared with the negative control group: #*p* < 0.05, ##*p* < 0.01, and ###*p* < 0.001.

In the murine infection model, mice in the infected control group exhibited acute weight loss, beginning within 24 h post-inoculation and culminating in a mean reduction of approximately 10 g by day 7. In contrast, mice treated with Nerol or MEM maintained stable body weight throughout the study and showed signs of gradual recovery over time (Figures 7A and 7B).

The bacterial load in the lungs of the model group was significantly higher than that in all treatment groups (*p* < 0.05; Figure 7C). The bacterial loads in the Nerol-H and MEM groups were comparable to those in the negative control group (*p* > 0.05), while the Nerol-M and Nerol-L groups exhibited similar efficacy. These *in vivo* findings indicate that Nerol effectively alleviates systemic lesions induced by *A. baumannii* infection.

### Nerol restores lung architecture and function post *A. baumannii* infection

Gross examination revealed severe hemorrhagic edema in the model group, characterized by diffusely dark-red discoloration, firm texture, and reduced elasticity of the lung lobes (Figure 7D). Histologically, hematoxylin and eosin (H&E) staining confirmed extensive lung injury, evidenced by alveolar flooding with erythrocytes and fibrin-rich exudates, disruption of alveolar septa, marked thickening of bronchiolar walls, and dense neutrophilic and lymphocytic infiltrates (Figure 7E).

Nerol treatment elicited a dose-dependent attenuation of these pathological features. Although focal hemorrhages and perivascular inflammatory aggregates persisted in the Nerol-M and Nerol-L groups, the Nerol-H displayed near-normal alveolar architecture with minimal exudate, similar to the negative control. The MEM group showed a comparable reduction in hemorrhage and inflammation. These results demonstrate that Nerol effectively mitigates histopathological damage and promotes structural recovery in a dose-dependent manner, highlighting its therapeutic potential against *A. baumannii*-induced pulmonary infection.

### The regulatory effect of Nerol on inflammatory mediators in mice infected with *A. baumannii*

**Figure 8:**
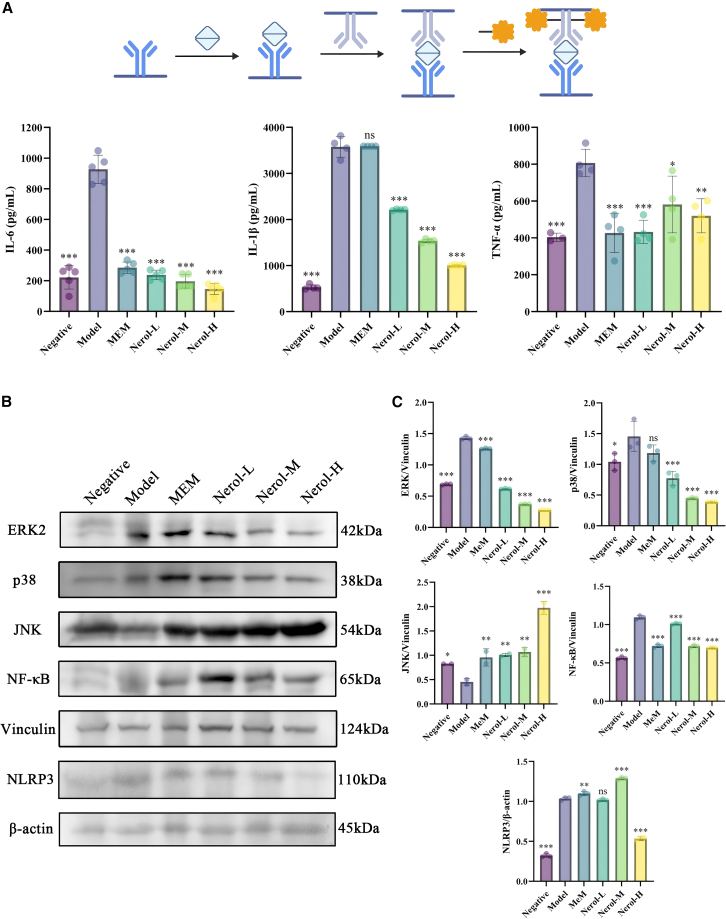
Effect of Nerol on inflammatory factors in mice (A) Quantitative analysis of inflammatory factors IL-6, IL-1β, and TNF-α in mouse lung tissues, the flowchart was created by https://biogdp.com/. (B) Immunoblotting of various proteins in lung tissues under different treatment conditions. (C) Reactive optical density analysis of p38, JNK, ERK, NLRP3, and NF-κB proteins in mouse lung tissues. Data are displayed as the means ± SD from three independent experiments, and significance was determined by one-way ANOVA with Dunnett’s post-test vs. Model: ∗*p* < 0.05, ∗∗*p* < 0.01, and ∗∗∗*p* < 0.001; ns, *p* ≥ 0.05.

Pro-inflammatory cytokines, including IL-1β, TNF-α, and IL-6, play critical roles in amplifying inflammatory cascades and contributing to tissue.^25^ Compared to the negative control, the model group exhibited a significant increase in the lung tissue levels of TNF-α (2.00-fold), IL-1β (6.75-fold), and IL-6 (4.18-fold), indicating a robust acute inflammatory response (*p* < 0.05, Figure 8A). Treatment with Nerol significantly reduced the levels of these pro-inflammatory cytokines in a dose-dependent manner compared to the model group (*p* < 0.05). The Nerol-H group showed the most pronounced effect, with cytokine levels reduced to 1.89- (TNF-α), 0.66- (IL-1β), and 1.29-fold (IL-6) of the negative control levels.

In *A. baumannii*-induced pneumonia, NF-κB activation and subsequent nuclear translocation initiate the transcription of pro-inflammatory genes, exacerbating the inflammatory response and tissue injury. Infection significantly activated the MAPK pathway, as evidenced by an increase in p38 (1.40-fold) and ERK (2.07-fold) in the model group compared to the negative control group (*p* < 0.05; Figures 8B and 8C). Simultaneously, NLRP3 inflammasome components increased 3.2-fold (*p* < 0.05). Nerol and MEM treatment significantly suppressed p38 and ERK expression, with Nerol exhibiting a dose-dependent effect. Notably, Nerol-H treatment reduced NLRP3 expression by 1.9-fold (*p* < 0.05), indicating that Nerol effectively inhibits proinflammatory factor release. Nerol and MEM treatments significantly suppressed p38 and ERK expression, with Nerol exhibiting a dose-dependent effect. In contrast, levels of JNK—another MAPK branch—were higher in all treatment groups than in the model group. The level of the cytosolic NF-κB p65 subunit was significantly higher in the model group than in the negative group, indicating NF-κB pathway activation and nuclear translocation. Both Nerol and MEM significantly inhibited NF-κB p65 nuclear accumulation (*p* < 0.05), with Nerol’s inhibitory effect being dose-dependent.

Nerol attenuates *A. baumannii*-induced acute pulmonary inflammation by dose-dependently suppressing the NF-κB/MAPK signaling cascade and subsequent pro-inflammatory mediator production.

## Discussion

Natural products have shown significant interest as promising inhibitors of *A. baumannii* resistance, owing to their ability to attenuate bacterial virulence, reduce environmental persistence, and exhibit favorable safety profiles. Perillaldehyde has been shown to markedly inhibit biofilm formation through the disruption of the bacterial cell membrane and interference with the QS system.^26^ Similarly, α-Pinene, another plant-derived compound, demonstrates potent anti-biofilm activity against *A. baumannii*.^27^ Nerol, perillaldehyde, and α-Pinene are all small-molecule compounds derived from plant essential oils. Among these, Nerol is a monoterpene alcohol that is also sourced from traditional Chinese medicines such as orange peel—and it is widely utilized in the food and pharmaceutical industries.^28^ Previous studies indicate that Nerol induces apoptosis in *Candida albicans* by compromising cell membrane integrity and permeability.^18^

The QS communication system, as a crucial regulator of clinical *A. baumannii* resistance, invasiveness and pathogenicity, prompts bacteria to adapt to environmental changes by modulating gene expression via signaling molecules. Among these, *abaI* and *abaR* participate in regulating the secretion of autoinductive signaling molecules AHLs, thereby controlling downstream gene expression involved in biofilm formation, pathogenicity, and other cellular processes. Otero et al.^29^ demonstrated the pivotal role of AHLs in quorum-sensing-mediated expression of virulence factors by inhibiting *A. baumannii* motility and biofilm formation with exogenous enzymes. This corroborates the present study’s finding that Nerol significantly inhibits *abaI*/*abaR*-mediated AHLs synthesis, reducing extracellular AHLs levels by 70%, thereby blocking QS signal amplification. Furthermore, Nerol downregulates the transcription of the BfmRS TCS’s *bfmS* and its response regulator *bfmR* (maximum reduction of 87%), thereby impairing type IV pili biosynthesis and subsequently inhibiting initial adhesion and three-dimensional structural maturation. To validate whether BfmS is a direct target of Nerol, we employed ITC to confirm the exothermic binding of Nerol to the BfmS sensor domain. This provides the first thermodynamic evidence demonstrating that Nerol blocks BfmS self-expression by occupying its binding pocket, thereby initiating downstream QS cascade inhibition. Correspondingly, standard strain ATCC 19606 and clinically resistant strains 5E9, 8D2, and 8E7 exhibited linear decreases in biofilm formation within the 1/4–1/2MBIC range, with maximum inhibition rates ≥60%. CFU counts confirmed viable bacterial counts ≥90%, ruling out non-specific bactericidal interference. Concentration-dependent inhibition was also observed in extracellular polymerization—the matrix component of the biofilm barrier—and in the twitching motility of *A. baumannii*. These phenotypic and molecular alterations closely resemble those reported in Xiong et al.,^30^ who demonstrated that *abaI* gene deletion inhibits biofilm formation and reduces pathogenicity in *A. baumannii* strain ATCC 19606. This anti-biofilm activity is similarly demonstrated in other small-molecule compounds. Findings by Tan et al.^31^ confirm that Farnesol exhibits dual efficacy in both bactericidal action and biofilm disruption. Samily et al.^32^ identified potent anti-biofilm potential in *p*-coumaric acid, which reverses *A. baumannii* resistance to MEM by interfering with virulence factors. In summary, Nerol downregulates the transcription of key QS system genes, disrupting AHLs-mediated signaling and the BfmRS signaling complex. This impedes pathogenic factor secretion and biofilm maturation, thereby diminishing *A. baumannii*’s virulence.

Subsequent proteomics data further confirmed that the differential protein expression profiles closely correlated with phenotypic observations. Specifically, key proteins OmpA and YiaD_1, which determine outer membrane abundance^33^ in *A. baumannii*, exhibited downregulated expression. This suggests impaired function in critical pathways such as virulence, membrane permeability, and antibiotic tolerance. Similarly, Nerol downregulated the expression of the histidine kinase BfmS, consistent with which BfmS-mediated biofilm formation and motility were significantly inhibited. This finding echoes the work of Abirami et al.,^9^ who reported that pyrogallol blocks antibiotic resistance and biofilm formation pathways in *A. baumannii* by downregulating key proteins, including BfmR and OmpA. EPS, as essential components of biofilm formation, are also critical determinants of drug tolerance. EPS synthesis and secretion are regulated by the bacterial Wza-Wzb-Wzc system, wherein the tyrosine kinase (Wzc) possesses autophosphorylation activity.^34^ The downregulation of Wzc in *A. baumannii* indicates that citral selectively affects EPS and proteins, thereby disrupting biofilm matrix formation.

Furthermore, Nerol downregulates the expression of efflux system proteins (RND)^35^ and ABC transporter family proteins (MacB),^36^^,^^37^ reducing the survival capacity of *A. baumannii* when exposed to residual concentrations of fungicides and rendering it more susceptible to attack by the host’s innate immune defense mechanisms. The inhibition of type II secretion system components (GspI and J512-2382) by Nerol represents another critical factor disrupting key pathways for pathogen effector delivery.^38^ The inhibition of T2SS blocks the secretion pathway for Sec-dependent virulence proteins (e.g., phospholipases and proteases), while the downregulation of MtgA and MurF^39^ disrupts the cross-linking of peptidoglycan tetrapeptide side chains. This leads to impaired cell wall osmotic equilibrium, ultimately exacerbating the osmotic damage inflicted by Nerol on bacterial cell membranes. The downregulation of the β-lactamase hydrolase-like protein (Blh-2) confirms that Nerol delays the development of antibiotic resistance in *A. baumannii* by reducing the activity of β-lactamases associated with drug resistance.^40^ These findings are highly consistent with the reports by Song et al. on the pivotal role of MacB in non-antibiotic resistance mechanisms during *A. baumannii* infections^36^ and the demonstration by Sobral et al. that MurF is indispensable for establishing resistance to β-lactam antibiotics.^41^

Moreover, the reduced expression of the large subunit protein bL28 (RpmB) indicates that its mRNA binding induces the translational termination of the bL28 operon, further reinforcing the bactericidal inhibitory effect of citral treatment on *A. baumannii*.

Protein-protein interaction network analysis identified five hub proteins that mediate processes such as outer membrane pore formation, biofilm formation, antibiotic resistance, protein transmembrane transport, and secretion complex assembly. The loss of their functions results in the inefficient secretion of pathogenic factors (such as lipooligosaccharides, polymers, and multidrug secretion substrates) from the cell surface, thereby reducing bacterial pathogenicity and antibiotic resistance. In summary, Nerol not only shares antimicrobial targets with other compounds but also exhibits a unique anti-virulence mechanism through a systematic multi-target intervention strategy.

*In vivo* experiments demonstrated that the administration of Nerol significantly inhibited the colonization of *A. baumannii* in mouse lungs and promoted the restoration of alveolar tissue structure. These findings that strongly support its anti-infective potential. Notably, effective anti-infective activity against bacterial pathogens typically involves not only the direct inhibition of bacterial growth or colonization but also the modulation of the host’s immune response to clear the infection,^42^ This connection between Nerol’s *in vivo* efficacy and host immunity provides context for understanding how it may interact with the body’s natural defense mechanisms against *A. baumannii*. However, excessive release of IL-6, TNF-α, and IL-1β can lead to systemic inflammatory responses.^43^ This study focused on the NF-κB/MAPK axis^44^ and found that Nerol significantly inhibited *A. baumannii*-induced NF-κB and p38/ERK levels, thereby downregulating downstream inflammatory expression. Meanwhile, elevated NLRP3 inflammasome levels in the lung tissue of the model group suggest its involvement in IL-1β maturation and release, indicating that Nerol effectively suppresses proinflammatory factor release by simultaneously blocking the NF-κB/MAPK and NLRP3 cascades. Previous reports have indicated that OmpA can promote IL-1β maturation and pro-inflammatory factor secretion by activating NF-κB, thereby exacerbating inflammation.^45^ This study observed that Nerol downregulates OmpA expression, which is positively correlated with NF-κB pathway inhibition, consistent with previous reports. Notably, total JNK abundance was elevated across all Nerol-treated groups relative to the model group, most likely reflecting enhanced protein synthesis and export of JNK monomers rather than kinase activation. As a key MAPK supporting NLRP3 inflammasome-mediated IL-1β secretion, JNK protein elevation did not translate into pro-inflammatory signaling: NLRP3 components decreased from 3.2-fold (model) to 1.9-fold (Nerol-H, *p* < 0.05), and IL-1β secretion returned to baseline levels, consistent with the overall anti-inflammatory phenotype. In summary, Nerol blocks NF-κB/MAPK signaling, reduces the release of TNF-α, IL-6, and IL-1β, thereby controlling infection while limiting host inflammatory damage, providing an intervention strategy for the treatment of *A. baumannii* pneumonia.

### Limitations of the study

This study conducted proteomic analysis as a single replicate to predict Nerol-target interactions in *A. baumannii*, identifying BfmS, MacB, YiaD_1, MurF, and MtgA as potential targets. Among these, BfmS was prioritized for further validation using orthogonal techniques, including RT-qPCR and ITC. However, several limitations should be acknowledged. First, molecular docking experiments used to predict protein-ligand interactions are inherently computational in nature and require further experimental validation to confirm the binding sites and affinities of AbaI, AbaR, and BfmR. Second, while BfmS was characterized at the transcriptional and biophysical levels, the downstream signaling cascade of the BfmRS TCS remains incompletely validated. Third, the broader protein network involved in BfmS-mediated quorum-sensing regulation, such as downstream effectors and compensatory pathways, was not fully explored. Fourth, proteomic screening was limited to a single reference strain (ATCC 19606), and the applicability of these protein-level findings to other clinically relevant strains or species warrants further investigation, despite the extension of transcriptional validation to multiple drug-resistant isolates (5E9, 8D2, 8E7). Finally, although NF-κB and MAPK pathway activation was inferred from gene expression data, direct evidence from phospho-specific antibodies and nuclear translocation assays was not provided. Future studies addressing these limitations will strengthen the mechanistic understanding of Nerol’s mode of action and its immunomodulatory potential.

## Resource availability

### Lead contact

Requests for further information and resources should be directed to and will be fulfilled by the lead contact, Hong Zeng (zenghong0705@163.com).

### Materials availability

The strains, experimental materials, and reagents used in this paper are listed in the Key Resources table. All unique/stable reagents in this study are available from the lead contact.

### Data and code availability

• Data availability: The mass spectrometry proteomics data have been deposited to the ProteomeXchange Consortium (https://proteomecentral.proteomexchange.org) via the iProX partner repository with the dataset identifier ProteomeXchange: PXD073438.
• Code availability: This article does not report original code.
• Other items: Any additional information required to reanalyze the data reported in this article is available from the lead contact upon request.

## Acknowledgments

This work was supported by the 10.13039/501100001809National Natural Science Foundation of China [grant no. 32560051] and the Guangxi Science and Technology Base and Talent Special Program [grant no. 2025GXNSFAA069977]. This study thanks BioGDP (https://biogdp.com/) for providing the online platform used to create the scientific illustrations in this manuscript.^46^

## Author contributions

Q.L.: writing – original draft, methodology, visualization, and supervision; S.W.: data curation and software; Y.S.: validation and resources; Z.L.: conceptualization; Y.L.: investigation; H.Z.: writing-review and editing, project administration, formal analysis, and funding acquisition.

## Declaration of interests

The authors declare no competing interests.

## STAR★Methods

### Key resources table

**Table:** 

REAGENT or RESOURCE	SOURCE	IDENTIFIER
**Antibodies**		

p38 MAPK Rabbit pAb	ABclonal, USA	Cat# A0227; RRID: AB_2757040
ERK2 Rabbit pAb	ABclonal, USA	Cat# A0229; RRID: AB_2757042
JNK Rabbit mAb	ABclonal, USA	Cat# A4867; RRID: AB_2863367
NF-κB p65/RelA Rabbit pAb	ABclonal, USA	Cat# A2547; RRID: AB_2764436
NLRP3 Rabbit pAb	ABclonal, USA	Cat# A21906; RRID: AB_3083447
β-Actin Rabbit pAb	ABclonal, USA	Cat# AC038; RRID: AB_2863784
Vinculin Rabbit pAb	ABclonal, USA	Cat# A14193; RRID: AB_2761053

**Bacterial and virus strains**		

*Acinetobacter baumannii*	ATCC, stored in the Zeng Laboratory at Youjiang Medical University for Nationalities	19606
*Acinetobacter baumannii*	Clinical isolates from the Affiliated Hospital of Youjiang Medical University for Nationalities	5E9, 8D2, 8E7, 5E1, 5E2, 5E6, 5F1, 5G5, 5G10, 5I3, 8D10
*Agrobacterium tumefaciens*	Stored in the Zeng Laboratory at Youjiang Medical University for Nationalities	KYC55
*Chromobacterium violaceum*	ATCC, stored in the Zeng Laboratory at Youjiang Medical University for Nationalities	12472
*Chromobacterium violaceum*	Stored in the Zeng Laboratory at Youjiang Medical University for Nationalities	CV026

**Chemicals, peptides, and recombinant proteins**		

Nerol	Sigma-Aldrich	CAS No. 106-25-2
Tween-80	Solarbio	T8360
O-nitrophenyl-β-D-galactopyranoside (ONPG)	Sigma	N1127-250 MG
5-Bromo-4-chloro-3-indolyl a-D-galactopyranoside	McLean	B885860
Yeast Powder Extract	OXOID	LP0021B
Pepsin	OXOID	LP0042B
Agar powder	Solarbio	A1890
Resazurin	Solarbio	CAS: 62758-13-8
Crystal violet	Solarbio	CAS: 548-62-9
Coomassie brilliant blue G-250	Solarbio	CAS: 6104-58-1
MHB	Binhe Microbiological Company	Cat#B162

**Critical commercial assays**		

Reverse Transcription Reagent SPAPK Script II	Spark	AG0305-B
2×SYBR Green qPCR Mix	Spark	AH0104-B
SPARK easy Rapid Bacterial RNA Extraction Kit	Spark	AC0401
Tumor Necrosis Factor-α (TNF-α) kit	Enzyme Immunoassay Biotechnology Co., Ltd.	MM-0132M1
Interleukin-1β (IL-1β) kit	Enzyme Immunoassay Biotechnology Co., Ltd.	MM-0040M1
Interleukin- 6 (IL-6) kit	Enzyme Immunoassay Biotechnology Co., Ltd.	MM-1011M1
Cell Counting Kit-8 (CCK-8)	Biosharp	BS350C

**Deposited data**		

Mass spectrometry proteomics data for *Acinetobacter baumannii*	ProteomeXchange Consortium (https://proteomecentral.proteomexchange.org)	ProteomeXchange: PXD073438

**Experimental models: Organisms/strains**		

BALB/c mice	Guangdong Vital River Laboratory Animal Technology Co., Ltd.	N/A

**Oligonucleotides**		

The primers used in this study are listed in Table S5	This research	N/A

**Software and algorithms**		

Prism version 10.2.3	GraphPad software	https://www.graphpad.com/
PyMOL 3.1	PyMOL	https://pymol.org/

### Experimental model and study participant details

Five-week-old male BALB/c mice, weighing 18–20 g, were purchased from Guangdong Vital River Laboratory Animal Technology Co., Ltd. Mice acclimated for 7 days in a controlled laboratory environment, provided with standard animal feed and free access to water. Environmental conditions maintained at 25 ± 3°C, 60% humidity, and a 12 h light-dark cycle. The Experimental Animal Management Committee of Youjiang Medical University for Nationalities in Baise, China (No.2024032802), approved all experimental procedures for toxicity assessment.

### Method details

#### Antibacterial activity

The MIC of Nerol against *A. baumannii* was determined using the micro-broth dilution method.^47^ Overnight *A. baumannii* cultures were adjusted to a concentration of 1.0 × 10^8^ CFU/mL, Nerol was co-incubated with an equal volume of bacterial suspension in sterile 96-well microtiter plates at 37°C, with a final concentration ranging from 0.002 to 1 mg/mL. Controls included a growth control without Nerol and a negative control containing only Luria-Bertani (LB) broth. After 24 h of incubation, absorbance at OD_600_ was measured, followed by the addition of 10 μL of resazurin dye (1 mg/mL) to each well and further incubation for 2 h at room temperature. The lowest antimicrobial concentration that limits the apparent growth of bacteria was defined as the MIC value.

#### Determination of the growth curve of *A. baumannii*

To evaluate the antibacterial potential of Nerol, this study monitored the growth trajectory of *A. baumannii* in LB medium supplemented with Nerol. Briefly, Nerol was dispensed into bacterial suspensions to achieve final Nerol levels of 1/4MIC and 1MIC; a drug-free control was included. Each culture was inoculated at 1.0 × 10^8^ CFU/mL and incubated at 37°C for 24 h. Growth was tracked every 2 h by measuring optical density at 600 nm using a UV-visible spectrophotometer.^48^

#### Pigment production

*C. violaceum* has emerged as a model organism for the study of bacterial communication in Gram-negative bacteria. A modified version of the method by Choo et al. was used to assess the inhibitory activity of Nerol on QS in *C. violaceum* ATCC 12472.^49^ *C. violaceum* was co-cultured with Nerol (final concentrations of 0.002–1 mg/mL) in 96-well plates at 30°C for 16 h. *C. violaceum* was centrifuged at 12,000 rpm for 10 min to precipitate *C. violaceum* and an equal volume of DMSO was added, centrifuged again, and the absorbance of the supernatant was measured at 585 nm.

#### AHLs production

In this study, *A. tumefaciens* KYC55 biosynthesis was used to screen for the production of AHLs.^50^ A suspension of *A. baumannii* was co-cultured with Nerol (1-1/4MIC) at 37°C for 8 h. The supernatant was separated by centrifugation at 12,000 rpm for 2 min. AHLs were extracted from the supernatant using ethyl acetate, air-dried at room temperature, and resuspended in distilled water to 1% of the volume of the original culture medium. AHLs were quantified by measuring β-galactosidase activity with the ONPG substrate, as described by John et al.^51^ *A. tumefaciens* KYC55 and AHLs were cultured in AT solution (Table S6). Z buffer, 0.05% SDS solution, chloroform and 4 mg/mL ONPG were added to the culture solution. 1M Na_2_CO_3_ was added to the samples after the solution had turned yellow and the time of discoloration was recorded as T (minutes) and the OD_420_ was measured. If no yellow coloration was seen, the test was stopped after 120 min. The relative activity of the AHLs was calculated according to the formula Miller units = (1,000×OD_420_)/(OD_600_×T×0.2). The extracted AHLs were mixed in LB medium and allowed to cool and solidify adding X-gal (40 μg/mL) as a color indicator. The *A. tumefaciens* KYC55 biosensor strain was then inoculated onto the prepared LB agar plates. Color changes in the biosensor strain were evaluated to determine a positive or negative response based on *A. tumefaciens* KYC55 activation.^52^

#### High-performance liquid chromatography (HPLC) validation of AHLs quantification

AHLs were extracted from *A. baumannii* supernatant using ethyl acetate as described above, dried, and resuspended in methanol. N-(3-Oxododecanoyl)-L-homoserine standards (0–0.1 mg/mL) were prepared and quantified via liquid chromatography-mass spectrometry (Ultimate 3000). Calculate AHLs inhibition rates and cross-validate with the KYC55 reporter gene.

#### Molecular docking

This study used molecular docking techniques to examine how two regulatory systems (AbaI/AbaR and BfmRS) regulate AHLs synthesis in *A. baumannii* QS. Predicted the binding properties of Nerol with key proteins (AbaR, AbaI, BfmR, and BfmS) in these systems. The structures of Nerol and the test proteins were downloaded from the PubChem Compound Database and the NCBI database. Proteins were prepared using AutoDock Tools and PyMOL, and molecular docking was performed using AutoDock Vina. Docking results were visualized and analyzed using Discovery Studio 2019 Client to generate 2D and 3D representations.

#### RT-qPCR

After co-culturing 1/2MIC Nerol with a suspension of *A. baumannii* at 37°C for 24 h, the collected bacteria were transferred to enzyme-free sterile test tubes. Total RNA was extracted using SPARKeasy Rapid Bacterial RNA Extraction Kit following the manufacturer’s instructions, treated with DNase I to remove genomic DNA. Reverse transcription was performed with SPAPK script II All-in-one RT SuperMix for RT-qPCR at 42°C for 15 min. RT-qPCR using SYBR Green Master Mix (10 μL reaction) with the following cycling conditions: 95°C for 5 min, 40 cycles of 95°C for 5 s and 60°C for 30 s. The 16S rRNA gene served as the internal reference. Each sample was run in triplicate, and relative expression levels were calculated using the 2^−ΔΔCt^ method.^53^ Primers used for RT-qPCR are listed in Table S5.

#### Biofilm formation

Biofilms, which are multicellular three-dimensional structures with close cell-to-cell contact, were quantified using crystal violet staining.^54^^,^^55^ A suspension of *A. baumannii* was incubated with Nerol for 48 h. The suspension was stained with 0.1% crystal violet dye for 30 min. Excess dye was rinsed off with sterile water, and then 95% ethanol was added to dissolve the crystal violet. Absorbance was measured at 570 nm to determine the MBIC. Biofilms cultured on glass coverslips within 6-well plates (Nerol 1-1/4MBIC) were fixed with 4% paraformaldehyde (15 min), stained with 0.1% crystal violet (5 min) and FITC-ConA (10 μg/mL, 30 min), and examined for morphology using an inverted microscope and CLSM.

#### Extracellular polymer content

Cultivate *A. baumannii* to a concentration of 1.0 × 10^8^ CFU/mL and incubate with an equal volume of LB medium containing different concentrations of Nerol (1-1/4MIC) for 24 h at 37°C. The culture medium was then centrifuged (12,000 rpm, 10 min, 4°C), and the supernatant was filtered through a 0.22 μm membrane. Coomassie Brilliant Blue G-250 was added to the supernatant for color development and absorbance was measured at 595 nm.^24^ A standard curve was constructed using human serum proteins to quantify the extracellular protein content of the samples.

The extracellular supernatant was mixed with anhydrous ethanol and left overnight, the precipitate was centrifuged and distilled water was added to obtain a polysaccharide solution, which was quantified using the phenol-sulfuric acid method.^56^ To this, 1 mL of 5% phenol and 5 mL of concentrated sulfuric acid were added to 1 mL of the polysaccharide solution, and the absorbance was measured at a wavelength of 490 nm.

#### Twitching motility

The effect of Nerol on the twitching motility of *A. baumannii* was assessed using LB medium containing 0.5% agar. The medium was cooled to 40°C, then different concentrations of Nerol (1-1/4MIC) were added and mixed thoroughly, and the untreated medium was used as a growth control.^57^ A suspension of *A. baumannii* and *C. violaceum*, adjusted to a concentration of 1.0 × 10^8^ CFU/mL, was inoculated onto the medium. The migration diameter of each strain across the plate was then measured.

#### Proteomics

Proteomics experiments were conducted on *A. baumannii* exposed to 0.25 mg/mL (1/2MIC) Nerol. Data quantification employed the Data-Independent Acquisition (DIA) mode, acquired via an Orbitrap Astral mass spectrometer. Analysis was conducted using DIA-NN version 1.9.2. Key software parameters were configured as follows: Trypsin was selected as the protease, with a maximum missing fragment count of 1. Fixed modifications included N-methylated (C), while dynamic modifications comprised oxidation (M) and acetylation (N-terminal). All reported data were based on a 99% protein identification confidence level, with False Discovery Rate (FDR) ≤ 1%. Protein intensities were normalised using the built-in quantification algorithm of the search software. Data underwent further filtering: proteins were retained if detected in ≥50% of any sample group. Missing values were imputed using the minimum value divided by two. Differential expression criteria were: Fold Change >2 or Fold Change <1/2, *p* value <0.05. The UniProt tool is the platform of choice for classifying differentially regulated proteins based on gene ontology (GO). To gain a deeper understanding of function, researchers used the KEGG reference pathway to understand the pathways that are altered in *A. baumannii in vivo*. To reveal the complex interactions between these differentially regulated proteins, the researchers used STRING version 12.0 and scored the interactions with medium confidence.^58^ The mass spectrometry proteomics data have been deposited to the ProteomeXchange Consortium (https://proteomecentral.proteomexchange.org) via the iProX partner repository^59^^,^^60^ with the dataset identifier PXD073438.

#### Isothermal titration calorimetry (ITC) analysis

The interaction between Nerol and BfmS was determined at 25°C using a Malvern ITC system equipped with MicroCal PEAQ-ITC control software. The pET22b (+) plasmid containing the BfmS gene was transformed into *E. coli* BL21(DE3), with the target protein being overexpressed and purified by BGI Shenzhen. Prior to experimentation, BfmS was diluted to 0.05 mmol/L in deionised water, while Nerol was prepared as a 1 mmol/L aqueous solution. During microinjection, 100 μL of Nerol solution was added to 80 μL of BfmS solution in 19 consecutive steps, each comprising 1 μL. Injection intervals were set at 150 s, with equilibrium periods of 300 s. The standard Microcal pEAQ-ITC control software package outputs standard interaction enthalpy (ΔH) and Gibbs free energy (ΔG), while also providing the reaction stoichiometry (n) and binding affinity constant (Ka).

#### *In vitro* and *in vivo* toxicity assessment

RAW264.7 cells (log phase, 1.0×10^4^ per well) were exposed to 0.25–4 mg/mL Nerol for 24 h, rinsed with PBS, and viability quantified by CCK-8 (450 nm). *In vivo*, mice received 30, 100 or 300 mg/kg Nerol (oral) for 5 days; liver and spleen were fixed in 4% formaldehyde, sectioned, and stained with H&E for histopathological assessment.^61^

#### *In vivo* efficacy

A mouse model of pneumonia was established following previously validated protocols.^26^ Briefly, mice were rendered immunocompromised via intraperitoneal injection of cyclophosphamide (40 mg/kg/day) for five consecutive days. Following anesthesia, pneumonia was induced by intranasal instillation of a 5E9 suspension of *A. baumannii* (100 μL, 1.0 × 10^8^ CFU/mL); negative control mice received 100 μL sterile PBS. Twenty-four hours post-inoculation, mice were monitored for successful modeling symptoms including dyspnea, reduced activity, and increased periorbital discharge. Following a 7-day pre-trial (*n* = 6 per group), oral doses of 30, 100, and 300 mg/kg Nerol were selected. The lowest dose (30 mg/kg) achieved approximately 0.25 mg/mL (1/2MIC) in bronchoalveolar lavage fluid. The higher two dose groups provided 2-fold and 6-fold safety margins, respectively, with no acute toxicity (normal behavior, body weight, and organ histology). Thus, mice were randomly assigned to six groups: Negative control, Model group, MEM group (4 mg/kg), low-dose Nerol group (30 mg/kg, Nerol-L), medium-dose Nerol group (100 mg/kg, Nerol-M), and high-dose Nerol group (300 mg/kg, Nerol-H). All doses were dissolved in 0.9% saline.

#### Bacterial load in lung tissue

A comprehensive assessment of the effect of Nerol on intrapulmonary *A. baumannii* load under aseptic conditions.^62^ At the end of drug treatment, mice were euthanized by cervical dislocation, and their lungs were excised. Lung tissues were homogenized and diluted in sterile saline. Then, 100 μL of each homogenate was plated on LB agar and incubated at 37°C for 24 h. Bacterial growth was recorded, and CFUs were calculated.

#### Histopathology

The severity of infection was assessed through histological examination of lung tissues. Lung samples were fixed in 4% formaldehyde, paraffin-embedded, sectioned, and stained with H&E. The liver and spleen were also examined for potential drug-induced pathological effects.

#### Enzyme-linked immunosorbent assay (ELISA)

Inflammatory markers were measured to assess the efficacy of Nerol in treating *A. baumannii*-induced inflammation. Lung tissue samples were homogenized in PBS, and the supernatant was collected and frozen. Levels of IL-6, IL-1β and TNF-α in lung tissue were quantified using ELISA kits.^63^

#### Western blot (WB)

RIPA lysis buffer and protease inhibitor (100:1) are added to the lung tissue, homogenized and lysed on ice for 30 min, centrifuged at 12,000 rpm for 20 min, and the supernatant is collected for determination of protein content. Adjust the sample concentration and heat at 95°C for 10 min in a water bath. Aliquots of protein were loaded into the wells of a 10% SDS-PAGE gel for electrophoresis, and the proteins were separated and transferred to a polyvinylidene difluoride (PVDF) membrane. The membrane was blocked with a blocking solution for 10 min and then incubated overnight at 4°C with primary antibody. Incubate the membrane with the appropriate dilution of secondary antibody for 1 h at room temperature. Capture chemiluminescent images using darkroom imaging technique ImageJ.

### Quantification and statistical analysis

Experimental data are presented as mean ± standard deviation (SD) and were analyzed using GraphPad Prism version 10.2.3. Statistical significance was assessed by one-way analysis of variance (ANOVA), with Dunnett’s test employed to examine associations between drug groups and the control group. Differences between the experimental groups were considered significant at a *p*-value of less than 0.05. Where ∗*p* < 0.05, ∗∗*p* < 0.01, ∗∗∗*p* < 0.001 indicated significance levels. All experiments were independently performed in triplicate.
