# Supplementary material for: Nerol as an anti-quorum sensing and therapeutic agent against Acinetobacter baumannii pneumonia
Source: iScience. 2026 Apr 13;29(5):115696. doi: 10.1016/j.isci.2026.115696 (PMC13127480; doi:10.1016/j.isci.2026.115696)
Supplement: Document S1. Figures S1–S5 and Tables S1–S6 [file mmc1.pdf]

iScience, Volume *29*

Supplemental information

Nerol as an anti-quorum sensing and therapeutic agent against *Acinetobacter baumannii* pneumonia

Qing Lu, Shuyun Wei, Yu Sun, Zhen Liang, Yulong Li, and Hong Zeng

Supplemental information
Supplemental Figures

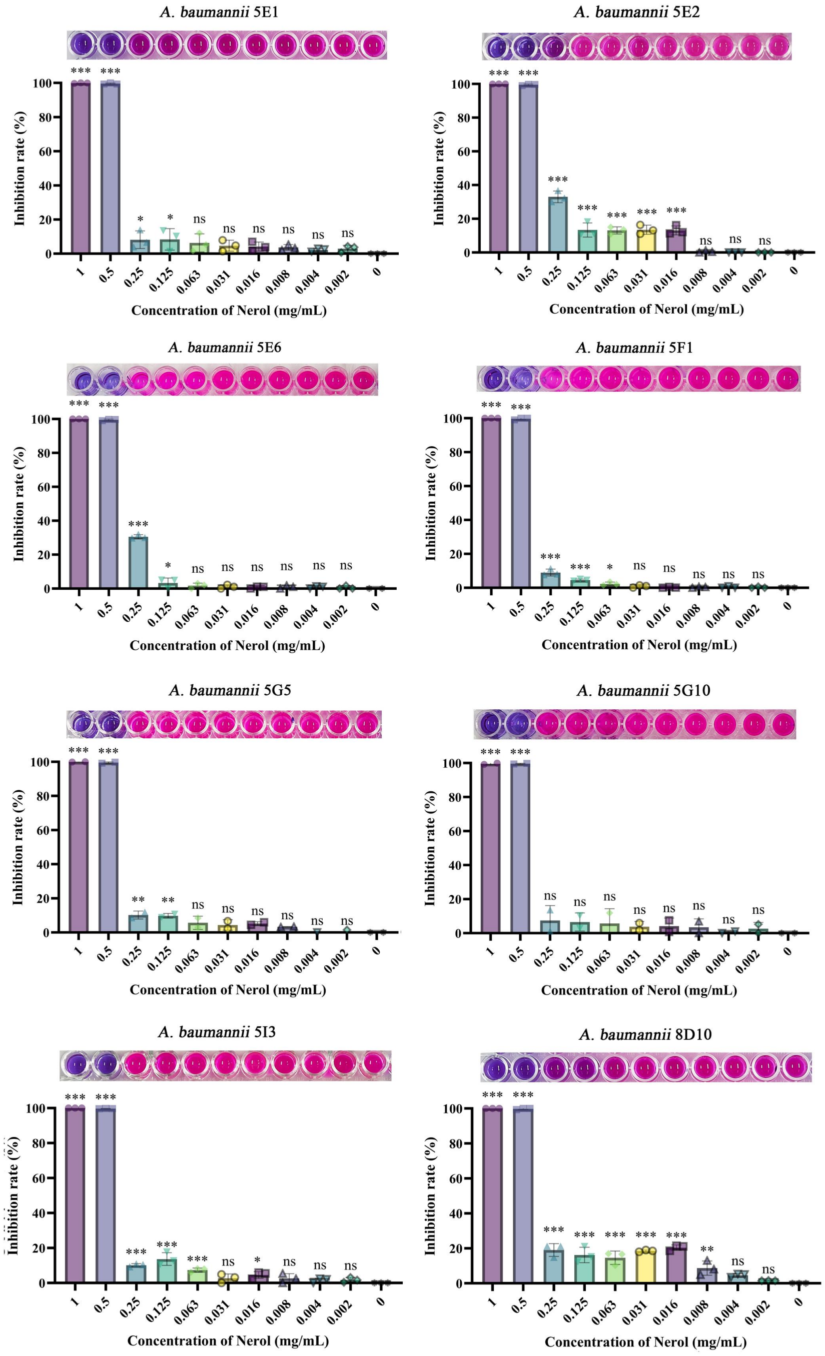

Figure S1. The MIC of Nerol against *A. baumannii*, related to Figure 1

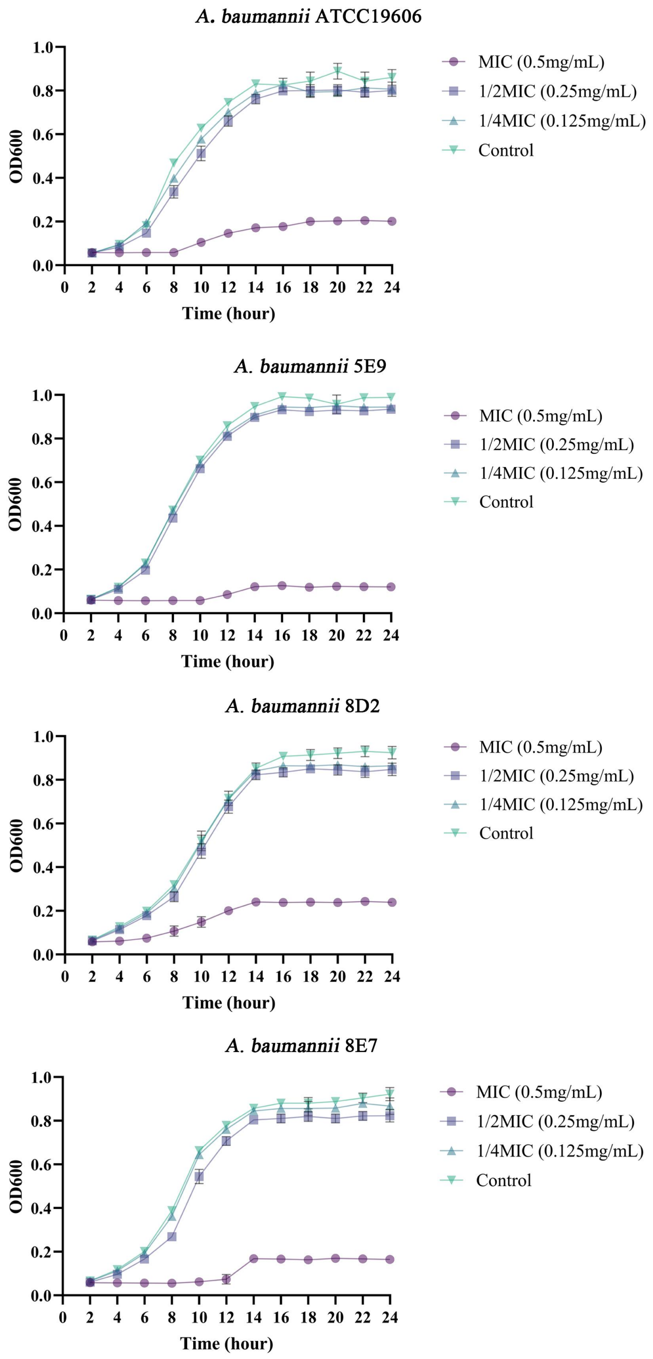

Figure S2. Effects of Nerol on the growth curve of *A. baumannii*

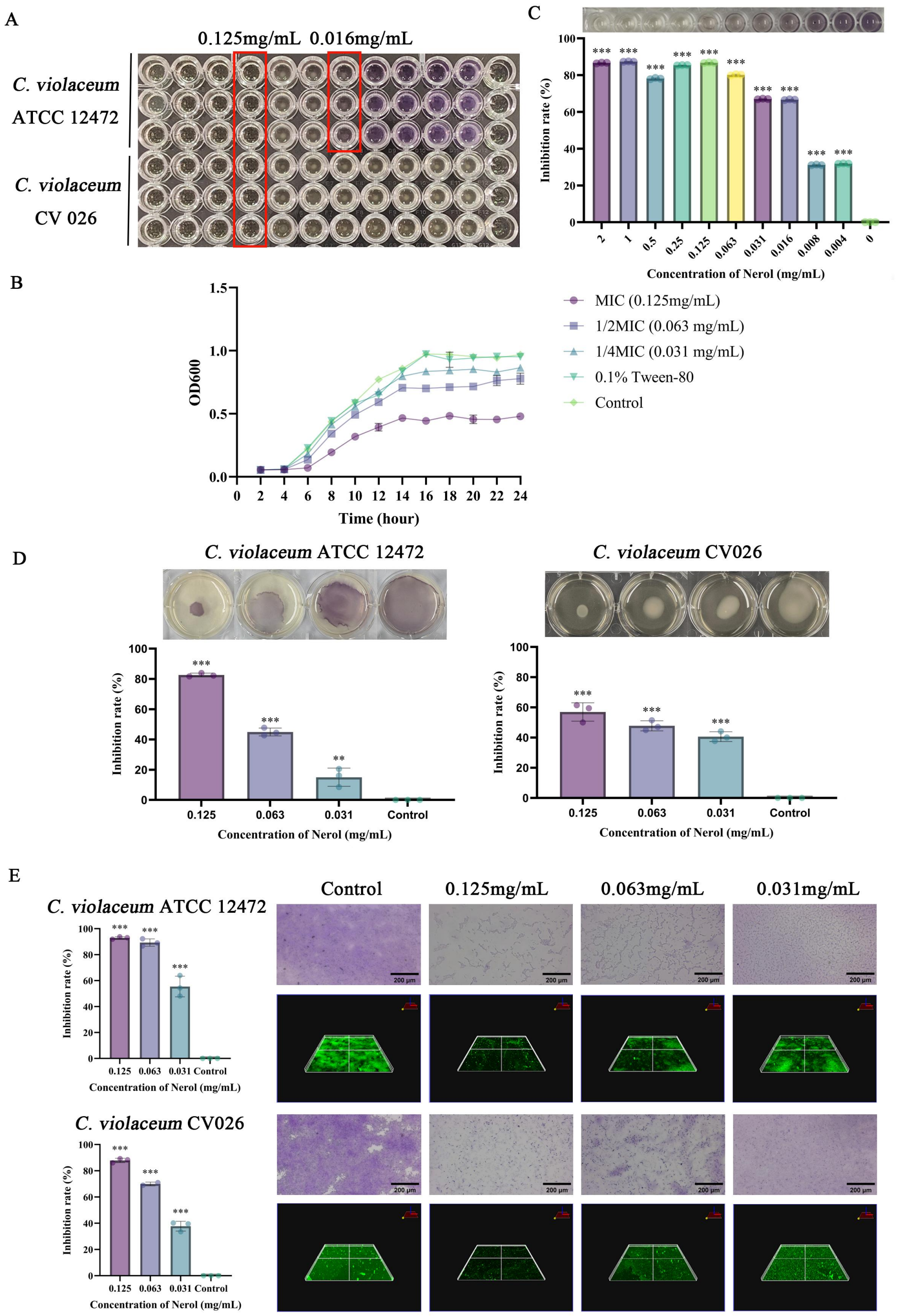

Figure S3. Inhibitory effect of Nerol on *C. violaceum*

(A) Minimum inhibitory concentration of Nerol on *C. violaceum*.

(B) Growth curve of *C. violaceum* in response to Nerol.

(C) Minimum inhibitory pigment concentration of Nerol against *C. violaceum*.

(D) Inhibitory effect of Nerol on the twitching motility of *C. violaceum*.

(E) Inhibitory effect of Nerol on the formation of biofilm of *C. violaceum*.

Data are displayed as the means ± SD from three independent experiments, and significance was determined by one-way ANOVA with Dunnett's post-test vs. Control: *p < 0.05, **p < 0.01, ***p < 0.001; ns, p ≥ 0.05.

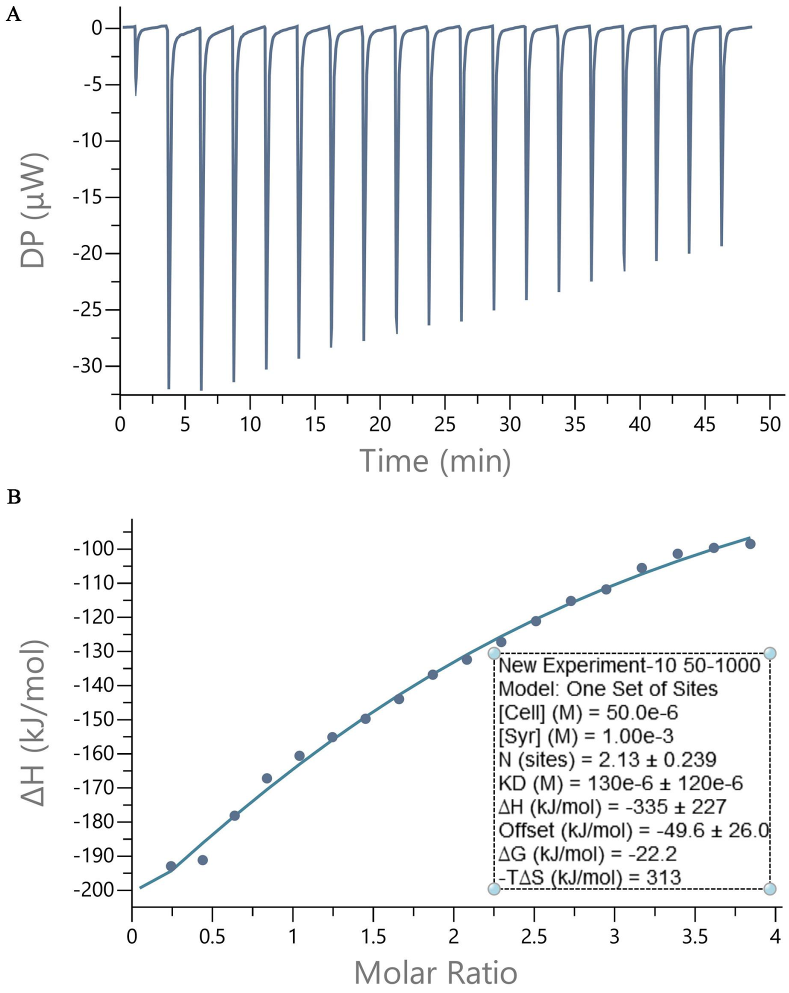

Figure S4. ITC analysis of Nerol binding to the BfmS sensor domain

(A) Representative raw ITC trace (upper panel) showing heat release upon injection of Nerol (1.00 mM) into BfmS (50.0 μM) at 25 °C.

(B) Integrated binding isotherm (lower panel) fitted to a one-site model; the stoichiometry (N) = 2.13 ± 0.24, dissociation constant (KD) = 130 ± 120 μM, enthalpy change (ΔH) = −335 ± 227 kJ/mol, and Gibbs free energy (ΔG) = −22.2 kJ/mol indicate a two-site, enthalpy-driven interaction that initiates downstream QS disruption.

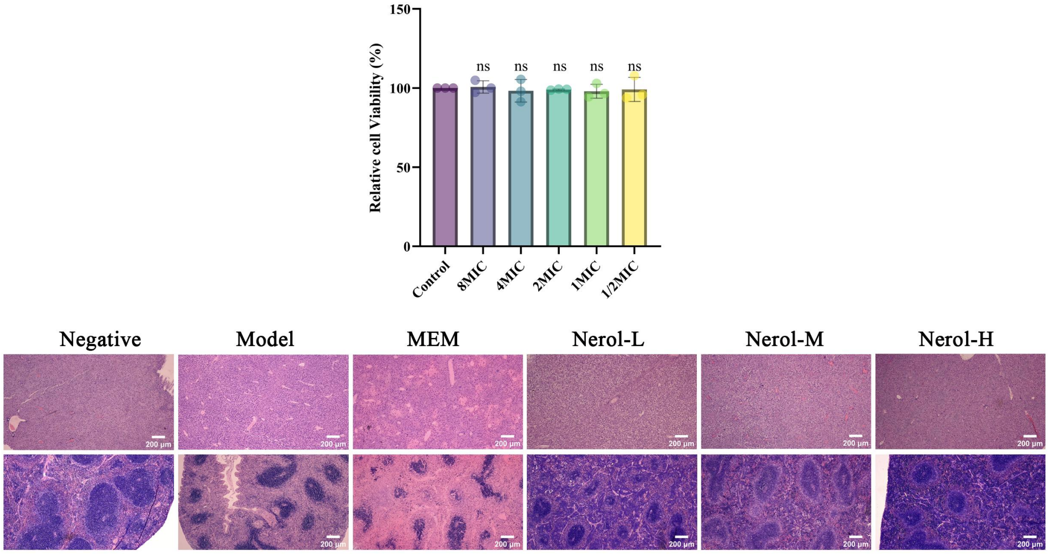

Figure S5. Safety evaluation of Nerol

Data are displayed as the means ± SD from three independent experiments, and significance was determined by one-way ANOVA with Dunnett's post-test vs. Control: *p < 0.05, **p < 0.01, ***p < 0.001; ns, $p \geq 0.05$.

Supplemental Tables

Table S1. Effect of 0.1% (v/v) Tween-80 on strain growth (mean ± SD, n=3, $p > 0.05$)

Bacterial Strain	Growth rate (%)
A. baumannii ATCC19606	98.78 ± 1.98 %
A. baumannii 5E9	96.73 ± 2.96 %
A. baumannii 8D2	98.49 ± 2.30 %
A. baumannii 8E7	97.77 ± 1.73 %

Table S2. MICs of 16 antibiotics agents against 3 clinical isolates of *A. baumannii*

Antibiotics		MIC(μg/mL)					
Categories	Drug	*A. baumannii* 5E9	R or S	*A. baumannii* 8D2	R or S	*A. baumannii* 8E7	R or S
Cephalosporins	Ceftazidime	>=64.0	R	>=64.0	R	>=64.0	R
	Cefepime	>=32.0	R	>=32.0	R	16	I
Carbapenems	Meropenem	>=16.0	R	>=16.0	R	>=16.0	R
	Imipenem	>=16.0	R	>=16.0	R	>=16.0	R
Quinolones	Ciprofloxacin	>=4.0	R	>=4.0	R	0.5	S
	Levofloxacin	>=8.0	R	>=8.0	R	0.25	S
Tetracyclines	Doxycycline	>=16.0	R	>=16.0	R	<=0.5	S
	Tigecycline	2	S	4	I	<=0.5	S
	Minocycline	>=16.0	R	>=16.0	R	<=1.0	S
Aminoglycosides	Tobramycin	>=16.0	R	>=16.0	R	2	S
β-lactam/β-lactamase inhibitor combinations	Aztreonam	>=64.0	R	>=64.0	R	32	I
	Cefoperazone/sulbactam	>=64.0	R	>=64.0	R	32	I
	Ticarcillin/Clavulanate	>=128	R	>=128	R	64	I
	Piperacillin/Tazobactam	>=128	R	>=128	R	>=128	R
Polymyxins	Polymyxin B	<=0.5	S	<=0.5	S	<=0.5	S
Sulfonamides	Paediatric Compound Sulfamethoxazole Tablets	>=320	R	>=320	R	>=320	R

R: Resistant, S: Susceptible, I: Intermediate.

Table S3. Quantitative detection of AHLs using HPLC

Sample	Peak area (%)	Concentration (mg/mL)
MIC (0.5 mg/mL)	0.38	0.058
1/2MIC (0.25 mg/mL)	1.58	0.22
1/4MIC (0.125mg/mL)	4.63	0.64
Control	7.65	1.05

Table S4. Biofilm formation ability of *A. baumannii*

Strains	Biofilm formation ability	Antibacterial resistance
A. baumannii ATCC 19606	Passive	N/A
A. baumannii 5E9	Strong positive	Ceftazidime, Cefepime, Meropenem, Imipenem, Ciprofloxacin, Levofloxacin, Doxycycline, Minocycline, Tobramycin Aztreonam, Cefoperazone/sulbactam, Ticarcillin/Clavulanate, Piperacillin/Tazobactam, Paediatric, Compound Sulfamethoxazole Tablets
A. baumannii 8D2	Strong positive	Ceftazidime, Cefepime, Meropenem, Imipenem, Ciprofloxacin, Levofloxacin, Doxycycline, Minocycline, Tobramycin Aztreonam, Cefoperazone/sulbactam, Ticarcillin/Clavulanate, Piperacillin/Tazobactam, Paediatric, Compound Sulfamethoxazole Tablets
A. baumannii 8E7	Strong positive	Ceftazidime, Meropenem, Imipenem, Piperacillin/Tazobactam, Paediatric, Compound Sulfamethoxazole Tablets

Table S5. Primers used in RT-qPCR

Primers	Sequence（5' to 3'）	Size (bp)
16S rRNA F	ACGGTCGCAAGACTAAAACTCA	108
16S rRNA R	GTATGTCAAGGCCAGGTAAGGT	108
abaI F	ATGCCTATTCCCTGCTCACC	87
abaI R	ATCGTCATTGGTGGACCTGC	87
abaR F	CGTTTCTGCCATGCTTCTGG	111
abaR R	CGCATCAAGGCTCGGATTTG	111
bfmS F	CCCCACCAAACCAGTAAGCA	130
bfmS R	ATTCCTGAGCAAGACCGCAA	130
bfmR F	CAGTACGCGCAGTCAACATT	118
bfmR R	GAGCAACCGGATCTTGTGGT	118

Table S6. Solution formulations for the detection of AHLs

Groups	Ingredient
AT solution	50mL 20×AT salt, 50mL 20×AT buffer, 10mL 50% (w/v) dextrose, 890mL H_2O
20×AT buffer	214g KH_2PO_4 (adjust pH to 7.3 using KOH), Volume to 1L
20×AT salt	40g $(NH_4)_2SO_4$, 1.56g $MgSO_4$, 0.152g $CaCl_2$, 0.1g $FeSO_4$*7H_2O, 0.044g $MnSO_4$*H_2O, Volume to 1L
Z Buffer	16.1g Na_2HPO_4*7H_2O, 5.5g NaH_2PO_4*H_2O, 0.75g KCl, 0.245g $MgSO_4$*7H_2O, 2.7mL β-mercaptoethanol, adjust pH to 7.0 with HCl, Volume to 1L
